# Supplementary material for: Implications of the Onset of Sweating on the Sweat Lactate Threshold
Source: Sensors (Basel). 2023 Mar 23;23(7):3378. doi: 10.3390/s23073378 (PMC10098635; doi:10.3390/s23073378)

**Figure S1.** Sweat lactate sensing device

This figure shows the sweat lactate sensor and the application device (Grace Imaging Inc., Tokyo, Japan). The sweat lactate data were recorded on a mobile application in real-time via a Bluetooth connection.

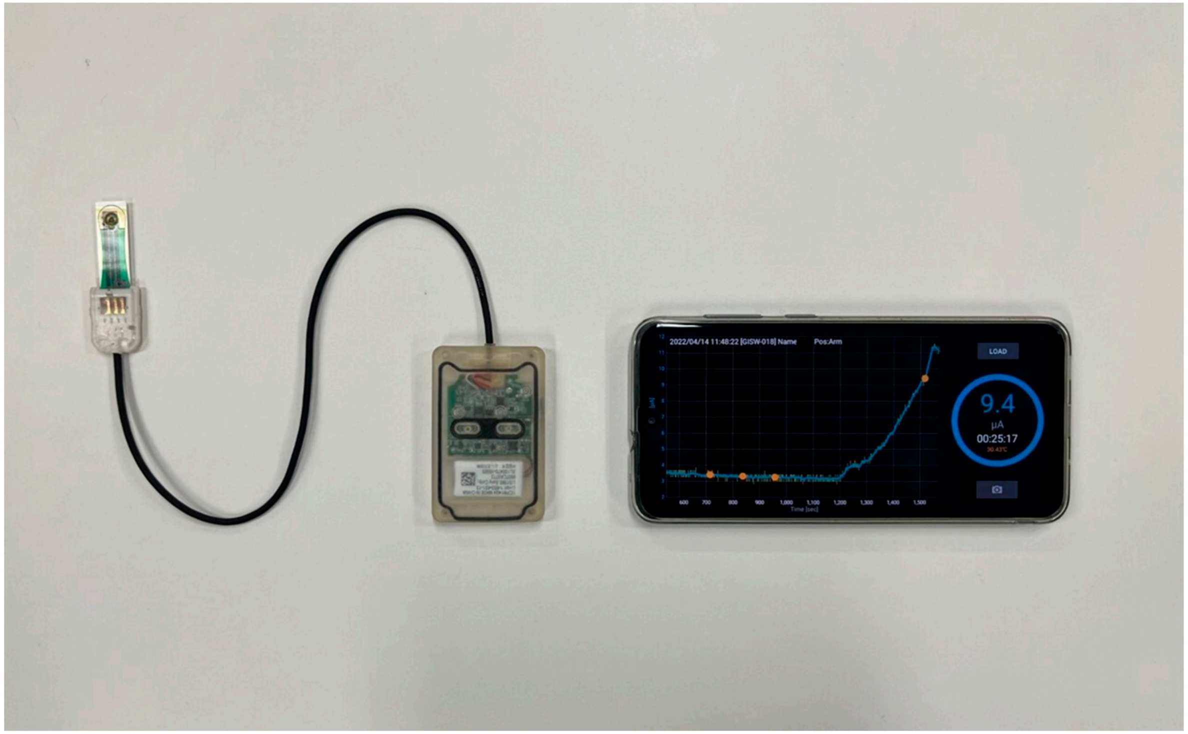

Supplement: Supplementary file 1 [file sensors-23-03378-s001.zip › sensors-2238251-supplementary.pdf]
